# Integrative Analyses Reveal *Tstd1* as a Potential Modulator of HDL Cholesterol and Mitochondrial Function in Mice

**DOI:** 10.3390/cells10112976

**Published:** 2021-11-01

**Authors:** Adi Zheng, Hao Li, Zhihui Feng, Jiankang Liu

**Affiliations:** 1 Department of Biomedical Sciences, University of Lausanne, Bugnon 7, 1005 Lausanne, Switzerland; adi.zheng@unil.ch; 2 Center for Mitochondrial Biology and Medicine, The Key Laboratory of Biomedical Information Engineering of Ministry of Education, School of Life Science and Technology, Xi’an Jiaotong University, Xi’an 710049, China; lihao.public@gmail.com; 3 University of Health and Rehabilitation Sciences, Qingdao 266071, China

**Keywords:** integrative analysis, HDL, quantitative trait loci (QTL), mediation analysis, transcriptome-wide association analysis (TWAS), correlation analysis, mitochondria, systems genetics

## Abstract

High-density lipoprotein (HDL) cholesterol levels are closely associated with human health and diseases. To identify genes modulating plasma HDL levels, we integrated HDL measurements and multi-omics data collected from diverse mouse cohorts and combined a list of systems genetics methods, including quantitative trait loci (QTL) mapping analysis, mediation analysis, transcriptome-wide association analysis (TWAS), and correlation analysis. We confirmed a significant and conserved QTL for plasma HDL on chromosome 1 and identified that *Tstd1* liver transcript correlates with plasma HDL in several independent mouse cohorts, suggesting *Tstd1* may be a potential modulator of plasma HDL levels. Correlation analysis using over 70 transcriptomics datasets in humans and mice revealed consistent correlations between *Tstd1* and genes known to be involved in cholesterol and HDL regulation. Consistent with strong enrichment in gene sets related to cholesterol and lipoproteins in the liver, mouse strains with high *Tstd1* exhibited higher plasma levels of HDL, total cholesterol and other lipid markers. GeneBridge using large-scale expression datasets identified conserved and positive associations between *TSTD1/Tstd1* and mitochondrial pathways, as well as cholesterol and lipid pathways in human, mouse and rat. In summary, we identified *Tstd1* as a new modulator of plasma HDL and mitochondrial function through integrative systems analyses, and proposed a new mechanism of HDL modulation and a potential therapeutic target for relevant diseases. This study highlights the value of such integrative approaches in revealing molecular mechanisms of complex traits or diseases.

## 1. Introduction 

High-density lipoprotein (HDL), a type of cholesterol with beneficial effects, possesses diverse anti-inflammatory, anti-oxidative and anti-apoptotic properties. The dysregulation of HDL is related to many diseases including metabolic diseases [1,2,3], cardiovascular diseases [4,5,6,7,8], cancer [9,10,11], and kidney diseases [12]. Raising HDL levels has been considered an effective strategy to lower the risk of related diseases [2,5,11]. However, the detailed mechanism underpinning HDL metabolism is still poorly understood. 

A mitochondrion is a dynamic organelle, and its dysfunction is related to many kinds of diseases [13,14,15]. HDL can help preserve mitochondrial structure and function [16], while genetic variants in mitochondrial DNA associate with HDL levels [17]. However, the intrinsic mechanism of the influence of mitochondria on HDL is unclear. Understanding the interplay between mitochondria and HDL will be useful for understanding the pathogenesis of relevant diseases.

The level of plasma HDL is a complex quantitative trait, which is controlled by many genes as well as external factors, such as diet [18] and exercise [19]. Quantitative trait loci (QTL) mapping is a common approach to identify chromosomal regions underlying complex traits, which has been used to identify the genes involved in the regulation of HDL levels [20]. A QTL is a genetic locus that associates with a quantitative phenotypic trait. Over 30 QTLs for HDL have been found in mice and humans. Interestingly, most QTLs for HDL levels in humans have homologous parts in mice, indicating a similar underlying mechanism of HDL modulation in humans and mice [20]. In addition, Gordon et al. showed that the protein diversity of the mouse plasma lipoproteome generally mirrors those in the human, supporting the use of mouse models for the studies of lipoprotein metabolism [21]. Therefore, the results from mouse models could potentially be used to infer results in humans [20,22,23]. Additionally, mouse models have proven to be valuable tools to increase our understanding of HDL metabolism, as well as its role in diseases such as atherosclerosis [24,25]. Knockout or transgenic mice have been widely used to evaluate the function of candidate genes in HDL regulation. Mouse genetics cohorts can also be used as additional resources to help interpret results from human genetic studies [26].

Many studies used different recombinant inbred mouse strains or F2 mice derived from two parental strains to study genetic loci associated with HDL levels [20,27,28,29]. Recently, outbred mouse populations were used to improve the resolution in mapping QTLs for HDL levels [30,31,32,33]. However, few genes underlying these QTLs have been identified due to the low recombination rate and mapping resolution of these cohorts [20,28]. Since QTL regions are usually quite broad and contain a long list of genes under the QTLs [34], narrowing down the candidate gene lists for QTLs is a great challenge in finally identifying causal genes that cause the variation of traits among individuals in cohorts. 

Previous studies revealed that the locus on distal chromosome 1 was responsible for major variations in plasma HDL levels in mice [20,23,27,28,32,35,36,37]. *Apoa2* was the first identified candidate gene under this QTL on chromosome 1 for HDL levels [35]. However, there are quite a large number of genes in this locus and most studies assumed *Apoa2* is the causal gene without testing others. Systemic studies using unbiased approaches integrating molecular data are needed to investigate other candidate genes in this locus. 

In the current study, we combined HDL measurements and transcriptomics data from established diversity outbred (DO) mice and other independent mouse cohorts and integrated a series of bioinformatic tools including mediation analysis, transcriptome-wide association analysis, and correlation analysis to identify candidate genes for the HDL QTL and identified *Tstd1* as a new potential HDL modulator. Our study revealed *Tstd1* as a new modulator of plasma HDL levels and mitochondrial function, and proposed a potential target for preventing and treating diseases related to HDL dysregulation. 

## 2. Materials and Methods

### 2.1. DO Datasets

Phenotypic trait, liver transcript, and genotype data for the diversity outbred (DO) mice were downloaded from the Diversity Outbred Database (https://do.jax.org/) on 21 August 2020 [30,33]. In summary, 835 mice were used in the study, of which HDL measurements are available for 783 mice, liver RNA-seq data are available for 478 mice, and liver proteomics data are available for 192 mice. 

QTL analysis was performed using the R/qtl2 package [38], and mediation analysis was performed using the R/Intermediate package (https://github.com/simecek/intermediate) on 21 May 2019 [39]. 

### 2.2. Data from Independent Mouse Cohorts

Data from 324 mice derived from the F2 cross between C57BL/6J and C3H/HeJ on ApoE null backgrounds were downloaded from genenetwork.org on 29 December 2020. All animals received a high-fat diet starting from 8 weeks of age. Mice were sacrificed at 24 weeks of age, and gene expression was measured from liver samples using microarray [40,41]. 

Data from 440 mice derived from the F2 cross between C57BL/6J and CAST/EiJ were downloaded from genenetwork.org on 29 December 2020. All mice were fed a high-fat Western diet starting from 10 weeks of age. Mice were sacrificed at 18 weeks of age, then liver gene expression was measured [42,43]. 

### 2.3. Transcriptome/Proteome-Wide Association Study (TWAS/PWAS)

TWAS/PWAS was performed by regressing plasma HDL levels on transcript or protein levels, while taking sex, diet, generation and the number of litters as covariates. The correction for multiple testing was performed using Bonferroni correction by taking into account the measured transcripts or proteins. 

### 2.4. Correlation Analysis

Statistical correlations between *Tstd1* expression in liver and plasma HDL levels as well as phenotypic traits were performed using Pearson’s product-moment correlation coefficient using the corAndPvalue function in the WGCNA package [44]. Correlations with *p*-values less than 0.05 were considered statistically significant. 

### 2.5. The Hybrid Mouse Diversity Panel (HMDP) Data and Extreme Strain Analysis

Liver transcriptome data from the HMDP mouse cohort was downloaded from GEO (GSE16780) [45,46]. The phenotypic measurements of the HMDP mice were downloaded from the Mouse Phenome Database (MPD) [47]. Differences in the phenotypic data between the *Tstd1*-high and *Tstd1*-low strains were evaluated using a Student’s *t*-test. The 10 strains with the highest and lowest *Tstd1* liver expression were used to calculate the fold-changes of gene expression in livers. Gene-set enrichment analysis (GSEA) was performed to identify the enriched gene sets between *Tstd1*-high and *Tstd1*-low strains using the R/fgsea package [48,49]. 

### 2.6. Correlation Heatmap

To identify the correlations between *Tstd1* and other genes, 34 human and 37 mouse liver transcriptomics datasets have been retrieved from GEO (listed in Supplementary Table S1). Genes in cholesterol and lipoprotein gene sets were obtained from Reactome [50]. Heatmaps illustrating the correlations between *Tstd1* expression and cholesterol/lipoprotein genes were generated using the heatmap.2 function in the R/gplots package. 

All statistical analyses and data visualizations in this study were carried out using R (version 4.0.4). 

## 3. Results

### 3.1. Influence of Sex and Diet on Plasma HDL Levels in Mice

**Figure 1:**
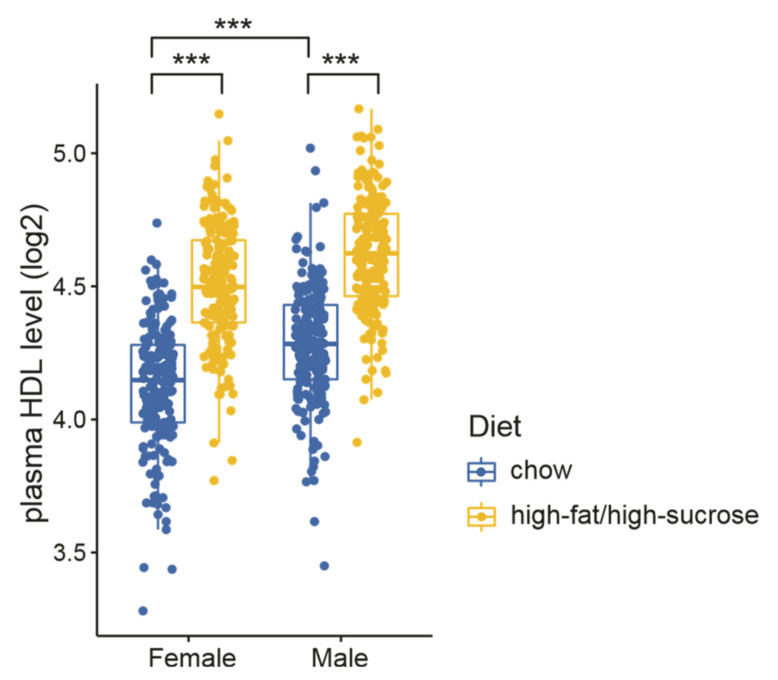
Influence of diet and sex on plasma HDL levels in a diversity outbred (DO) mouse cohort. Plasma HDLs were measured for 835 mice at 8 weeks of age. Statistical significance between different groups was determined by two-way ANOVA. ***, *p* < 0.001.

To investigate the potential genes modulating plasma HDL, we re-analyzed the HDL measurements from a recently established DO mouse cohort containing 835 animals (half were females and half were males) [30]. Mice were fed either chow or a high-fat/high-sucrose diet (HFHS) starting from weaning. Plasma HDL levels were measured at 8 weeks of age. As expected, sex and diet showed a strong influence on plasma HDL levels—males had higher HDL than females regardless of the diets, and mice fed with HFHS had higher HDLs than those on a normal diet (Figure 1). The increased HDL levels induced by HFHS feeding are consistent with other studies [51,52,53], probably as a defensive response to transport the increased lipids by HFHS.

**Figure 2:**
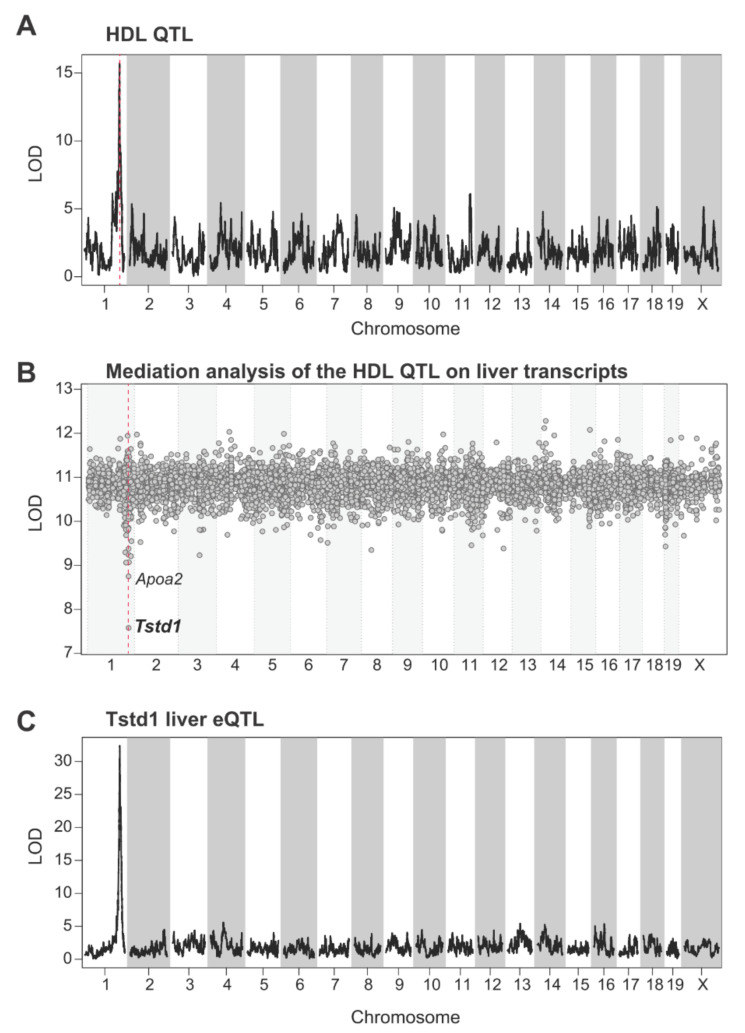
QTL and mediation analysis revealed *Tstd1* as a potential modulator for plasma HDL levels in the DO mouse cohort. (**A**) QTL plot showed the genome-wide linkage of plasma HDL levels in the DO mouse cohort. Chromosomes are represented across the x-axis and the significance of QTLs (log of odds, LOD) is represented on the y-axis. The vertical red dashed line indicates the QTL peak on chromosome 1. (**B**) Mediation analysis identified the possible mediators for the HDL QTL on chromosome 1. Genes are plotted across the x-axis based on their genetic positions on chromosomes. The conditional QTL scores for all genes in the mediation analysis are represented on the y-axis. The vertical red dashed line indicates the QTL peak for HDL on chromosome 1. (**C**) eQTL plot for *Tstd1* in the liver of the DO mouse cohort. *Tstd1* mapped a cis-eQTL at the same locus of the QTL for HDL levels.

We then mapped the QTL for plasma HDL levels using R/qtl2 with sex and diet as covariates [38], and found a significant and conserved QTL on chromosome 1 with the peak at 171.29 Mb (Figure 2A). This QTL has previously been discovered by several independent studies using different mouse cohorts [20,23,33,35,36], and *Apoa2*, a gene encoding a protein component of HDL, is commonly believed to be the causal gene in this locus [23,35]. However, there are many other genes in this locus that could also be of interest and relevant to the modulation of HDL, although studies investigating the involvement of these genes on HDL are not yet available [23]. 

To identify the candidate genes regulating HDL levels in an unbiased manner, we applied mediation analysis by integrating the HDL measurements from the DO mice with transcriptome data obtained from the same mouse cohort. Mediation analysis uses the expression levels of each gene individually as additive covariates to estimate the possibility that these genes are causal mediators of the physiological trait QTLs [39]. Including the expression of the mediator in the QTL mapping model should significantly decrease the QTL effect and, therefore, show a decrease in the log of the odds (LOD) score. Through mediation analysis, we found a few genes under the HDL QTL on chromosome 1 that reduced the LOD score of the HDL QTL when taken as covariates, including genes known to be relevant to the regulation of HDLs. In particular, *Tstd1* stood out as the best candidate gene for the HDL QTL from the mediation analysis, while the known HDL-related gene *Apoa2* was much less significant (Figure 2B). *Tstd1* colocalizes with the HDL QTL on chromosome 1 and had a significant *cis*-eQTL (expression QTL) in the liver at the same locus (Figure 2C). 

### 3.2. Transcriptome- and Proteome-Wide Associations Verifies the Association between Tstd1 and HDL

**Figure 3:**
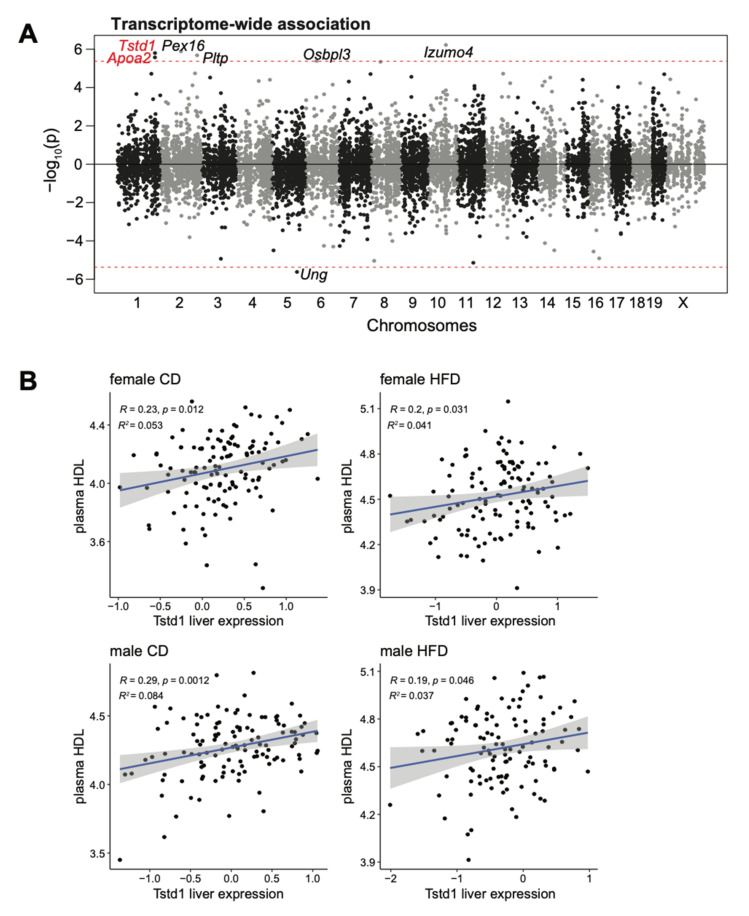
Transcriptome-wide association study (TWAS) further confirmed the association between the plasma HDL and *Tstd1*. (**A**) Manhattan plot depicting the associations of transcripts with positive effects (increased expression leads to higher HDL levels) and transcripts with negative effects (increased expression leads to lower HDLs). Transcripts were plotted across the chromosomes with x-axis as the chromosome coordinates and y-axis as the significance of the association. The threshold for transcriptome-wide significance was determined based on the number of transcripts tested (*p* <  4.2  ×  10^−6^, red dashed line). (**B**) Correlations between *Tstd1* liver expression and plasma HDL levels in the DO mouse cohort. Individual animals were divided into four groups based on their sex and diet conditions. The Pearson’s correlation coefficient and significance were indicated.

We then used TWAS to identify genes that strongly correlate with plasma HDL levels by controlling the intrinsic and external factors including sex and diet, and identified seven genes (*Izumo4*, *Pex16*, *Tstd1*, *Pltp*, *Apoa2*, *Ung*, and *Osbpl3*) at the transcriptome-wide significance (Figure 3A). Of these seven genes, *Tstd1* and *Apoa2* located under the HDL QTL on distal chromosome 1, with *Tstd1* (*p* = 1.59  ×  10^−6^), had a higher transcriptome-wide association than *Apoa2* (*p* = 2.68  ×  10^−6^). Indeed, the *Tstd1* liver transcript was consistently, although mildly, correlated with plasma HDL levels in both male and female mice fed with either diet (Figure 3B). 

**Figure 4:**
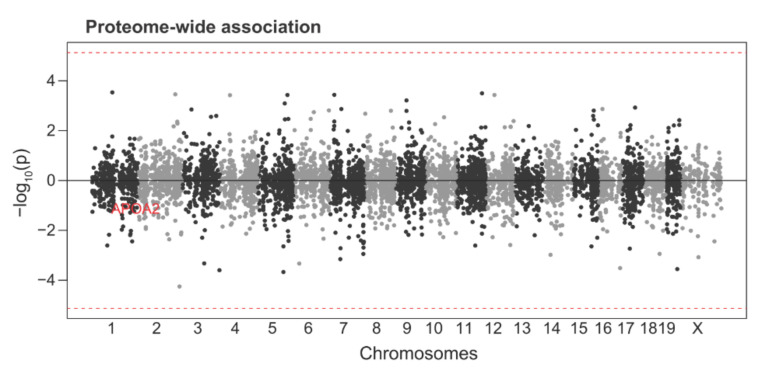
Proteome-wide association study (PWAS) revealed a non-significant association between the plasma HDL and APOA2. Manhattan plot depicting the associations of proteins with positive effects (increased expression leads to higher HDL levels) and proteins with negative effects (increased expression leads to lower HDLs). Proteins were plotted across the chromosomes with x-axis as the chromosome coordinates and y-axis as the significance of the association. The threshold for proteome-wide significance was determined based on the number of proteins tested (*p* <  7.4  ×  10^−6^, red dashed line).

Since proteomics data is available for 6740 proteins in the DO mice, we also performed proteome-wide association analysis (PWAS) to reveal the proteins associated with HDL levels in an unbiased manner. The protein measurements of APOA2 but not TSTD1 are available in the proteomics data. However, there were no associated proteins with HDLs at the proteome-wide significance, and APOA2 showed even slightly negative associations with HDL (Figure 4), which is in contrast to the results from TWAS in Figure 3. 

In summary, *Tstd1* stood out as the best candidate gene for the HDL QTL on chromosome 1 based on results from the mediation analysis, TWAS and PWAS. 

### 3.3. Confirmation of the Tstd1-HDL Connection Using Independent Data Sets

**Figure 5:**
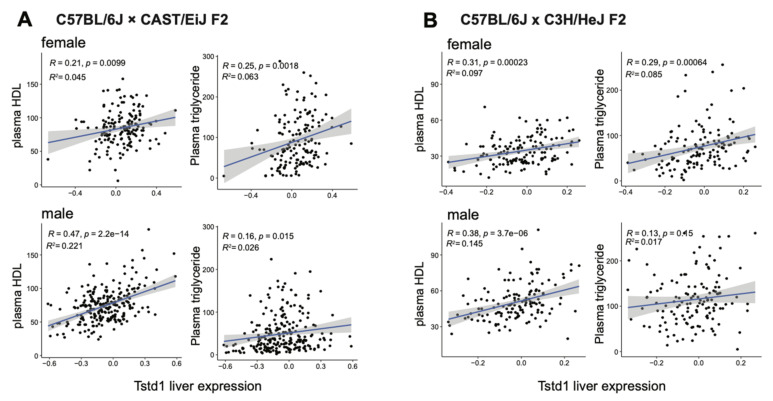
Validation of the link between *Tstd1* and HDLs using independent mouse cohorts. Correlations between liver *Tstd1* expression and plasma HDL and triglyceride levels were investigated in male and female mice from the F2 cohorts derived from C57BL/6J and CAST/EiJ (**A**) and C57BL/6J and C3H/HeJ (**B**) Pearson’s correlation coefficients and *p*-values were indicated.

To support our findings from the DO mouse cohort, we validated the connection between *Tstd1* and HDL levels using data from other independent mouse studies. As shown in Figure 5A, hepatic *Tstd1* levels positively associated with plasma HDL levels in both male and female mice in an F2 cohort derived from C57BL/6J and CAST/EiJ [43]. The result could be further confirmed using another independent F2 mouse cohort derived from C57BL/6J and C3H/HeJ (Figure 5B) [41,54]. These results confirmed the conserved association between *Tstd1* and HDL levels across mouse populations. 

### 3.4. Tstd1 Correlates with Genes Involved in HDL and Cholesterol Synthesis

**Figure 6:**
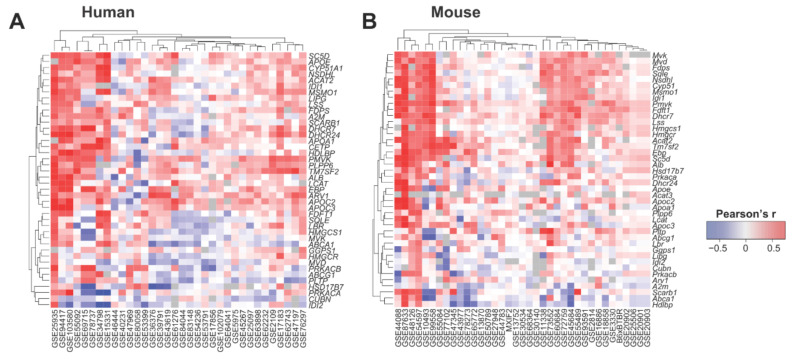
*Tstd1* co-expressed with key genes involved in HDL and cholesterol synthesis in human and mouse. Heatmap showing the correlations between *TSTD1/Tstd1* and key HDL and cholesterol genes in 34 and 37 liver transcriptome datasets from human (**A**) and mouse (**B**), respectively. Pearson’s correlation coefficients are indicated in the heatmap.

We then tested if the liver expression of *TSTD1/Tstd1* correlated with key genes known to be relevant for HDL and cholesterol synthesis using 34 and 37 liver transcriptome datasets obtained from human and mouse, respectively. Indeed, we found consistent positive correlations between *TSTD1/Tstd1* and relevant genes in HDL and cholesterol synthesis (Figure 6, Tables S1–S3), suggesting that *Tstd1* could influence HDL levels through modulating the genes involved in the molecular processes of cholesterol and HDL synthesis. 

### 3.5. Role of Tstd1 on Lipid Markers and Liver Transcript Profiles in Mice 

**Figure 7:**
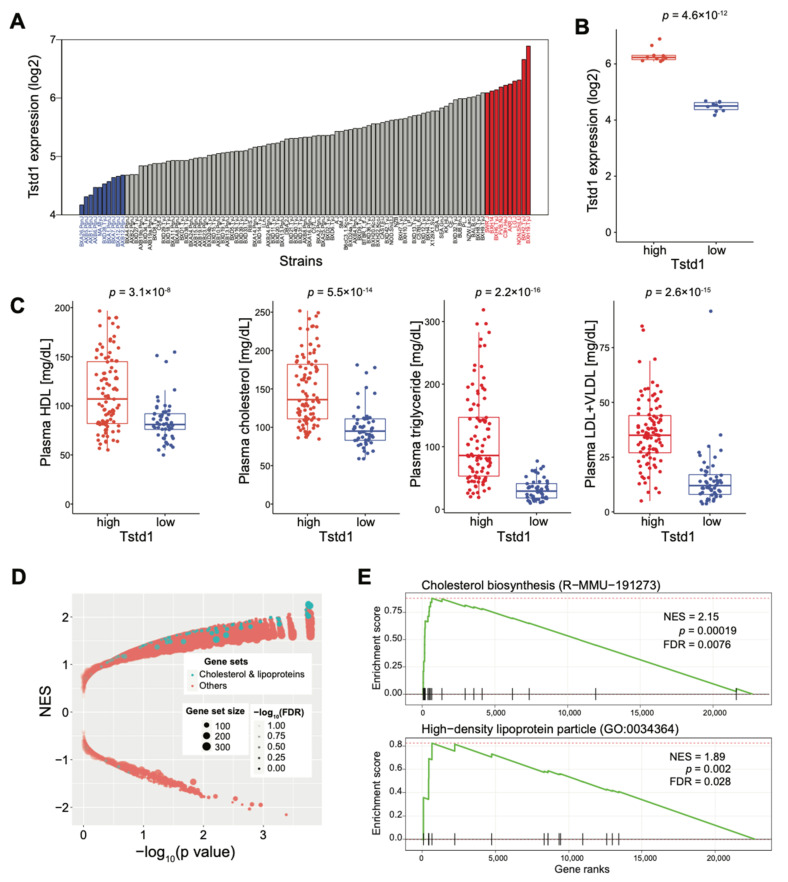
Impact of *Tstd1* on lipid levels in the HMDP mouse population. (**A**) The expression of hepatic *Tstd1* in the mouse strains at 16 weeks of age. The 10 *Tstd1*-high (red) or *Tstd1*-low (blue) strains with the highest and lowest hepatic *Tstd1* expression levels in the HMDP population, respectively. (**B**) The *Tstd1* liver expression in the *Tstd1*-high and *Tstd1*-low strains. (**C**) The lipid levels of individual animals from the *Tstd1*-high and *Tstd1*-low strains. Data are represented as mean ± SEM. Significance was determined by Student’s *t*-test. (**D**) Gene set enrichment analysis (GSEA) results featured the expression changes of genes involved in cholesterol and lipoprotein relevant pathways (highlighted in blue dots) between the *Tstd1*-high and *Tstd1*-low strains. NES: normalized enrichment score. FDR: false discovery rate. (**E**) Enrichment plots showing the enrichment of genes in the Cholesterol biosynthesis (upper) and HDL particle (lower) gene sets.

To further explore the link between *Tstd1* and HDL, we examined the effect of *Tstd1* in lipid-related markers in the Hybrid Mouse Diversity Panel (HMDP) mouse population, which is composed of approximately 100 well-characterized inbred strains [45,55,56]. We first checked the expression variation of *Tstd1* in the livers of 99 strains of the HDMP cohort, and observed 6.6-fold differences across the HMDP strains (Figure 7A). We then used 10 strains with the highest or lowest hepatic *Tstd1* expression to estimate the differences between lipid-related markers and expression patterns. There was a significant difference between 10 strains with low (blue) and 10 strains with high (red) *Tstd1* expression in the livers (Figure 7B). HDL levels were significantly higher in strains with higher *Tstd1* expression, as well as other lipid-related markers in the plasma, including total cholesterol, TG, and LDL + VLDL (Figure 7C). We compared the liver gene expression differences between *Tstd1*-high and *Tstd1*-low strains and performed gene set enrichment analysis (GSEA) to identify the enriched gene sets between the two groups [49]. We found that gene sets related to cholesterol and lipoproteins, for example, cholesterol biosynthesis and HDL particle gene sets were strongly enriched (Figure 7D,E). These data further confirmed the involvement of *Tstd1* in cholesterol and HDL metabolism and a possible mechanism of *Tstd1* in modulating HDL levels.

### 3.6. Tstd1 Associates with Cholesterol and Mitochondrial Pathways

**Figure 8:**
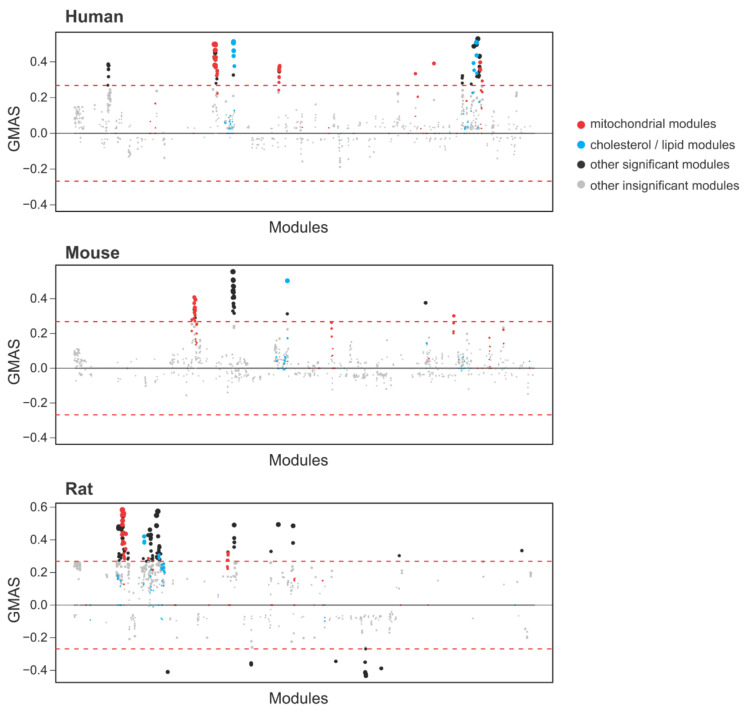
GeneBridge analysis identified the associations between *TSTD1/Tstd1* and cholesterol metabolism as well as mitochondrial function. GeneBridge analysis was performed using liver datasets in human, mouse and rat. Pathway modules were arranged horizontally according to the similarity between modules, and the gene-module association scores (GMAS) were shown in the y-axis. The significance thresholds are indicated by the red dashed line. Modules related to mitochondrial function are indicated by red dots, while those related to cholesterol or lipid metabolism are indicated by blue dots.

To identify the potential molecular functions of *Tstd1*, we applied the gene-module association determination (G-MAD) analysis in the GeneBridge toolkit (https://www.systems-genetics.org/genebridge/) on 25 March 2021 [57]. Liver expression data from 42 human datasets, 34 mouse datasets, and 27 rat datasets were used in the analysis. Consistently, we observed positive associations between *TSTD1/Tstd1* and pathways related to cholesterol or lipid metabolism, validating our findings that *Tstd1* modulates HDL levels (Figure 8, Tables S4–S6). In addition, we found that *TSTD1/Tstd1* is significantly associated with mitochondrial pathways in human, mouse and rat, demonstrating the involvement of *Tstd1* in regulating mitochondrial functions. 

## 4. Discussion

HDL has been considered protective against many kinds of diseases, including metabolic diseases [1,2,3], cardiovascular diseases [4,5,6,7,8], and cancer [9,10,11]. More than 30 QTLs for HDL have been discovered in humans and mice [20]. However, identifying the causative genes based on only QTL information is challenging, because the number of chromosome recombination events is limited and the QTL regions are normally very broad and include a large number of genes. Most QTLs are located in the noncoding region of the genome, suggesting that these loci influence phenotypic traits by affecting gene expressions (so-called expression QTLs, or eQTLs) [58]. Integration of gene expression information helps to identify genes underlying QTL that are responsible for variances of the phenotypic traits. Many studies using mouse genetic populations identified a significant and conserved QTL on distal chromosome 1 for plasma HDL levels. Though there are many genes in this locus, *Apoa2* has been commonly believed to be the causal gene for this QTL [20,23,27,28,32,35,36,37]. However, very limited efforts have been devoted to studying the possible involvement of other genes in this locus on HDL. 

Through an unbiased mediation approach, we identified that *Tstd1* from this region could be a candidate gene regulating HDL levels. *Tstd1* has a *cis*-eQTL in the liver, which is colocalized with the HDL QTL, suggesting a link between *Tstd1* and plasma HDL levels in mice. We further performed TWAS analysis and verified the connection between *Tstd1* and plasma HDL. Notably, the association of *Tstd1* to HDL is stronger than that of the known HDL-related gene *Apoa2*. PWAS analysis showed no significant association between liver APOA2 protein levels and plasma HDL. Therefore, these results suggest *Tstd1* is a candidate gene for the HDL QTL on chromosome 1 in the DO cohort. 

To substantiate our hypothesis, we further tested whether the expression differences in hepatic *Tstd1* transcripts were associated with variations in plasma HDL and triglyceride levels using the correlation analysis. The results showed consistent positive correlations between *Tstd1* liver transcripts and HDL levels regardless of sex and diet conditions, indicating overexpression of *Tstd1* could increase HDL levels. In addition, we confirmed the positive association between *Tstd1* and HDL using several independent mouse cohorts, including F2 mouse populations derived from C57BL/6J and CAST/EiJ, and C57BL/6J and C3H/HeJ, respectively. It should be noted that although the correlation between *Tstd1* hepatic expression and HDL levels are consistently discovered across several independent mouse cohorts in both genders, *Tstd1* expression only explains a small proportion of the total variance of HDL levels. 

At the molecular level, *Tstd1* correlated with genes involved in cholesterol and lipoprotein modulation, suggesting a potential molecular mechanism of *Tstd1* on HDL. We examined the role of *Tstd1* on lipid markers and liver profiles using strains from the HMDP cohort expressing high or low *Tstd1* levels, and confirmed the involvement of *Tstd1* in cholesterol metabolism. Mouse strains with high *Tstd1* expression in the liver exhibited increased plasma HDL and cholesterol levels, supporting the potential role of *Tstd1* in modulating HDL. Furthermore, *Tstd1*-high strains showed increased enriched gene sets related to cholesterol and lipoproteins, suggesting that *Tstd1* regulates HDL by affecting genes involved in molecular processes for HDL metabolism. 

GeneBridge analysis using large-scale expression datasets confirmed the conserved association between *TSTD1/Tstd1* and cholesterol or lipid metabolism in human, mouse and rat, and predicted the involvement of *TSTD1/Tstd1* in modulating mitochondrial functions. The crosstalk among *Tstd1*, mitochondria and HDL needs to be further explored. *Tstd1* could be a potential linker between mitochondrial function and HDL modulation. 

We noticed that *Tstd1* was not detected in many earlier versions of microarrays, which could be one of the main reasons why its role in modulating HDL has not been well illustrated. *Tstd1* encodes a putative thiosulfate:glutathione sulfurtransferase, but the understanding of its function is greatly limited. Further experiments to evaluate the role of *Tstd1* in regulating HDL levels and metabolic status are warranted. Furthermore, validation of the *TSTD1*-HDL connection in humans is needed. In addition, *Tstd1* showed conserved association with cholesterol and mitochondrial pathways, and the underlying mechanism remains to be studied. 

In summary, we identified *Tstd1* as a novel candidate gene regulating plasma HDL levels and mitochondrial function through integrative analyses, which could provide a potential therapeutic target for relevant diseases. Our study provides an efficient model for the identification of candidate genes involved in complex traits such as HDL levels. 

## Data Availability

Data was obtained and are available from Gene Expression Omnibus (https://www.ncbi.nlm.nih.gov/geo/), Diversity Outbred Database (https://do.jax.org/), Mouse Phenome Database (https://phenome.jax.org/), GeneBridge (https://www.systems-genetics.org/genebridge/), and GeneNetwork (http://genenetwork.org/).

## Supplementary Materials

The following are available online at https://www.mdpi.com/article/10.3390/cells10112976/s1, Table S1. List of derived datasets derived from human or mouse liver used in this study. Table S2. Correlation coefficients between *TSTD1* and all genes in 34 human liver transcriptomics datasets. Table S3. Correlation coefficients between *Tstd1* and all genes in 37 mouse liver transcriptomics datasets. Table S4. G-MAD results for *TSTD1* using 42 human liver expression datasets. Table S5. G-MAD results for *Tstd1* using 34 mouse liver expression datasets. Table S6. G-MAD results for *Tstd1* using 27 rat liver expression datasets.
